# Bibliometric Analysis of the Role of Artificial Intelligence in Detecting Maxillofacial Fractures

**DOI:** 10.7759/cureus.75630

**Published:** 2024-12-13

**Authors:** Bovaz Babu, Divya Vinayachandran, Ganesh C, Shanthi M, Krithika CL

**Affiliations:** 1 Oral Medicine and Radiology, SRM Kattankulathur Dental College and Hospital, SRM Institute of Science and Technology (SRMIST), Chennai, IND; 2 Oral Medicine and Radiology, SRM Dental College Ramapuram, SRM Institute of Science and Technology (SRMIST), Chennai, IND

**Keywords:** artificial intelligence in radiology, computer-assisted diagnosis, deep learning, facial bone fractures, maxillofacial trauma

## Abstract

Facial bone fractures are a common occurrence in trauma cases, particularly in India where road traffic accidents contribute significantly. Over the past few years, artificial intelligence (AI) has become a potent instrument to help medical professionals diagnose and treat facial fractures. This study aims to perform a bibliometric analysis, that is, a quantitative and qualitative analysis, of publications focusing on the role of AI in detecting facial bone fractures. Bibliometric analysis can be a very strong measure of research productivity and analysis of trends within a given area of research. Data were drawn from the Dimensions AI database; 58 relevant scientific articles were analyzed in this study. This bibliometric analysis aims to assess the volume of research in this area, identifying key trends, authors, institutions, and countries contributing to the literature. The Dimensions AI database was used to gather and analyze relevant data, shedding light on the research impact through indicators such as the h-index, citation counts, and publication trends. This review will depict the landscape of research work, highlighting the rising influence of the use of AI for accurate diagnostics in facial bone fractures and further detailing gaps and potential avenues of future research directed toward solutions such as standardizing datasets and clinical integration.

## Introduction and background

Fractures in the maxillofacial region can cause extreme facial deformities and functional issues in the jaw, strongly influencing a person's general life, social life, and quality of life. Complications associated with this may include emotional stress, economic factors, and psychological problems. In extreme cases, fractures lead to critical conditions like injuries to the brain, breathing issues, heavy loss of blood, or damage to the eyes and spine because the position of the jaw creates unique anatomical characteristics. In India, maxillofacial injuries are a significant public health concern, particularly due to the high rate of road traffic accidents. Timely and accurately diagnosing such fractures is crucial to ensure proper treatment and reduce the risk of long-term complications. Such fractures permanently disturb the development of the facial structure in children. Because of the differences in causes and treatment in different regions, patterns of such fractures need to be identified to improve clinical practice in diagnosis, treatment, and prevention [1].

The rapid advancement of information technology leading to the widespread adoption of artificial intelligence (AI) especially in healthcare and radiology significantly enhances diagnostic accuracy, predicts outcomes, and assesses risks by processing large datasets with exceptional speed and precision. In radiology, AI is slowly becoming an essential tool for analyzing medical images from computed tomography (CT) scans, magnetic resonance imaging (MRI), and X-rays. It accurately identifies and recognizes fracture lines and detects early signs of disease, which the human eye could miss out on at certain times. This advantage not only improves diagnostic accuracy but also reduces the time required for image interpretation, thereby accelerating clinical decision-making. AI excels in managing vast volumes of data and uncovering patterns that may be overlooked by human observers. For instance, in cases of maxillofacial injuries, AI effectively analyzes radiographic data to identify trends and predict complications while also generating 3D reconstructions that enhance treatment planning. To successfully integrate AI into clinical workflows, several challenges must be addressed, including the necessity for large, annotated datasets and robust healthcare infrastructure. Additionally, practical barriers such as the need for extensive training and concerns regarding the reliability of AI outputs and patient confidentiality warrant careful consideration. In summary, AI holds great potential to transform diagnostic accuracy and efficiency in radiology. By confronting these technical and practical challenges, along with advancing technologies and collaborative efforts to standardize datasets and provide training, we can fully realize AI’s transformative impact in medicine [2].

This analysis examines the current literature on the applications of AI in the detection of facial bone fractures. Its objective is to clarify the state of this technology by mapping the research landscape, investigating publication patterns from prominent contributors, and identifying emerging trends. Utilizing Dimension AI as the primary database, this bibliometric analysis explores trends, key contributors, and research gaps related to the application of AI in maxillofacial trauma, focusing on publications from the past decade (2014-2024).

AI-powered diagnostic tools allow for rapid fracture detection within seconds through the use of helical CT or MRI scans, which is vital for addressing the complex anatomical structures involved in maxillofacial injuries. This paper aims to analyze publication trends, as well as geographic and institutional contributions, while also highlighting existing research gaps in the application of AI for detecting maxillofacial injuries. A thorough review of the literature will deepen our understanding of the progress achieved thus far and pinpoint areas that warrant further investigation in future research.

## Review

Methodology

Bibliometric analysis is an effective tool for gaining insights into research trends and patterns across specific fields. By quantitatively assessing publication data, this method offers valuable insights into research impact, scholarly productivity, and collaborative relationships among institutions and researchers. In this study, we conducted an extensive analysis of data sourced from the Dimensions AI database, selected for its comprehensive coverage of high-impact journals in the field. Our focus was on the evolving landscape of AI with facial bone fractures. We identified 58 relevant articles, underscoring the increasing interest and advancements in this area of research.

Data extraction

The primary source of data for this analysis was the Dimensions AI database, chosen for its repository of scientific literature, including high-impact journals. Additionally, supplementary searches were conducted in other databases, such as PubMed, MDPI, Springer Link, BMJ Open, Frontiers, IOS Press, and Nature, to ensure comprehensive coverage. Keywords like "artificial intelligence", "machine learning", "deep learning", "facial bone fracture", and "maxillofacial trauma" were used to retrieve relevant publications. Articles, reviews, and conference papers from the last decade were included in the study. The inclusion criteria for this study specify that it must focus on AI applications in maxillofacial trauma care and include only peer-reviewed articles, reviews, and conference papers published between 2013 and 2024. Meanwhile, the exclusion criteria consist of duplicate publications, preprints, and studies that are unrelated to AI in healthcare or maxillofacial trauma. Additionally, any non-peer-reviewed papers or studies not published in English will also be excluded. The limitations of Dimensions AI may result in the exclusion of certain regional or non-indexed publications, potentially diminishing the overall comprehensiveness of the analysis. Consequently, research from less dominant geographic regions or smaller institutions may not be adequately represented. Additionally, the database's focus on indexing peer-reviewed articles may restrict the inclusion of gray literature, such as conference proceedings and technical reports.

Data analysis 

VOSviewer (Centre for Science and Technology Studies, Leiden University, The Netherlands) and Biblioshiny (RStudio (Posit PBC, Boston, MA, US)) were used to explore and visualize the bibliometric data. VOSviewer produced network visualizations including co-authorship networks and keyword co-occurrence maps, revealing collaboration patterns and research focus areas. Biblioshiny integrated with RStudio provided advanced bibliometric metrics, such as citation analysis, geographic trends, and historical publication patterns. These tools complemented each other, making it possible to give an integrated interpretation of bibliometric trends, unearthing the main contributors, and mapping the evolving landscape of AI research in maxillofacial trauma care [3,4]. This approach provided a comprehensive understanding of emerging trends, key contributors, and gaps in the field.

Risk of bias assessment

With reliance placed on the Dimensions AI database for primary analysis, bias exists in such an approach: it depends mainly on the indexing of peer-reviewed articles while excluding other forms of literature, and it excludes studies made regionally. The imposition of using predefined keywords to be found may ignore the use of appropriate terms. This could potentially result in linguistic and selection bias because only articles published in English and peer-reviewed are considered, which would exclude significant contributions from regions where other languages are predominant and emerging research in preprints. In addition, the visualization tools VOSviewer and Biblioshiny are user-defined, which may have implications for the objectivity of the network and trend analysis.

Results

Most Globally Cited Documents

**Figure 1:**
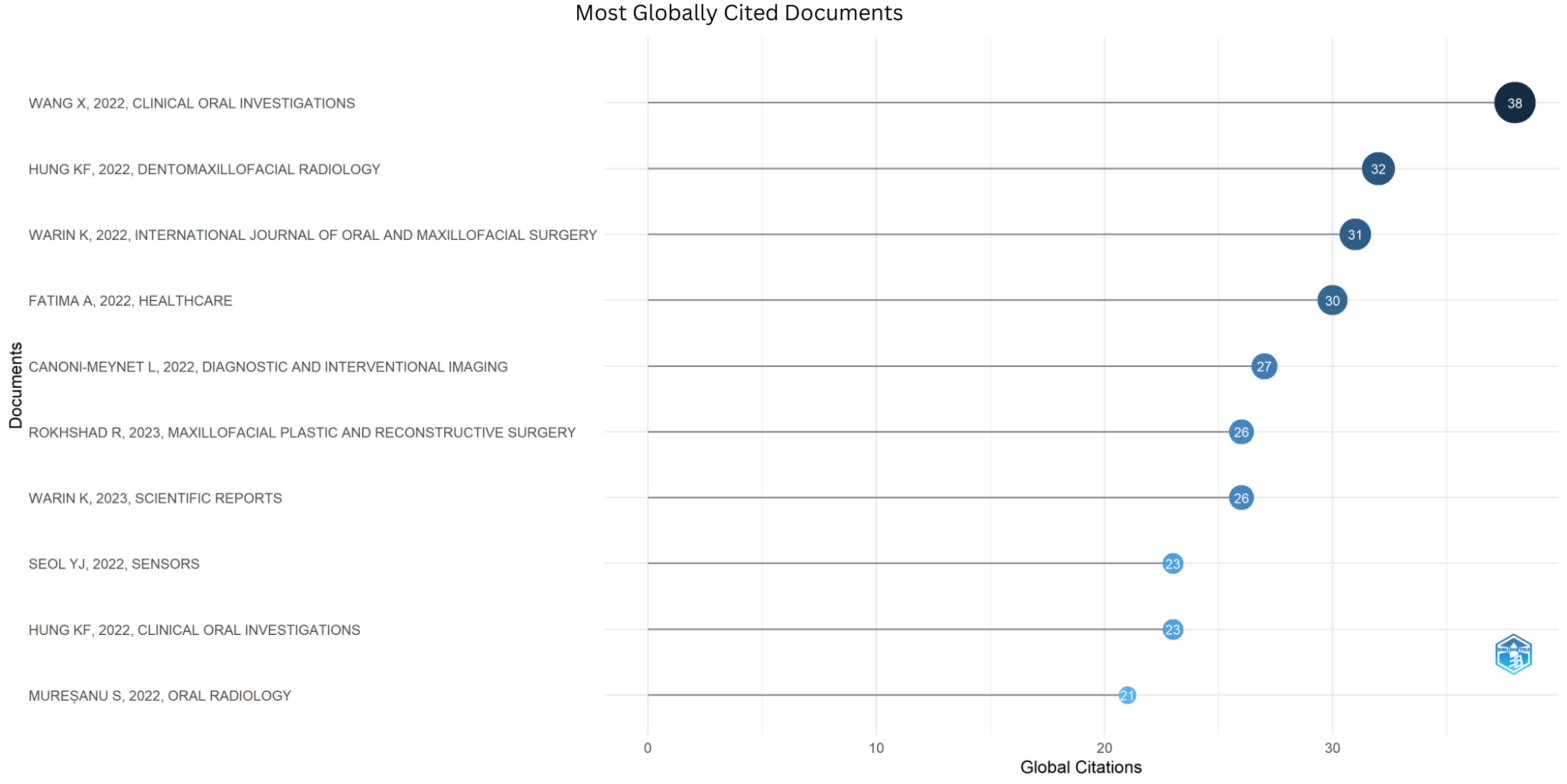
Most Globally Cited Documents References [5-14]

Globally cited documents in the field show the broader impact of AI technologies on facial trauma management. The most cited article was by Wang et al., "Detection and classification of mandibular fracture on CT scan using a deep convolutional neural network" (Table 1, Figure 1).

**Table 1:** Most Globally Cited Documents TC: total number of citations

Paper	Journal	DOI	Title	Total citations	TC per year	Normalized TC
Wang et al., 2022 [5]	Clinical Oral Investigations	10.1007/s00784-022-04427-8	Detection and classification of mandibular fracture on CT scan using deep convolutional neural network	38	12.67	2.13
Hung et al., 2022 [6]	Dentomaxillofacial Radiology	10.1259/dmfr.20220335	Personalized dental medicine, artificial intelligence, and their relevance for dentomaxillofacial imaging	32	10.67	1.79
Warin et al., 2022 [7]	International Journal of Oral and Maxillofacial Surgery	10.1016/j.ijom.2022.03.056	Assessment of deep convolutional neural network models for mandibular fracture detection in panoramic radiographs	31	10.33	1.74
Fatima et al., 2022 [8]	Healthcare	10.3390/healthcare10112188	Advancements in dentistry with artificial intelligence: current clinical applications and future perspectives	30	10.00	1.68
Canoni-Meynet et al., 2022 [9]	Diagnostic and Interventional Imaging	10.1016/j.diii.2022.06.004	Added value of an artificial intelligence solution for fracture detection in the radiologist's daily trauma emergencies workflow	27	9.00	1.51
Rokhshad et al., 2023 [10]	Maxillofacial Plastic and Reconstructive Surgery	10.1186/s40902-023-00382-w	Artificial intelligence applications and ethical challenges in oral and maxillofacial cosmetic surgery: a narrative review	26	13.00	4.24
Warin et al., 2023 [11]	Scientific Reports	10.1038/s41598-023-30640-w	Maxillofacial fracture detection and classification in computed tomography images using convolutional neural network-based models	26	13.00	4.24
Seol et al., 2022 [12]	Sensors	10.3390/s22020506	A study on 3D deep learning-based automatic diagnosis of nasal fractures	23	7.67	1.29
Hung et al., 2022 [13]	Clinical Oral Investigations	10.1007/s00784-022-04477-y	Potential and impact of artificial intelligence algorithms in dento-maxillofacial radiology	23	7.67	1.29
Mureșanu et al., 2022 [14]	Oral Radiology	10.1007/s11282-022-00660-9	Artificial intelligence models for clinical usage in dentistry with a focus on dentomaxillofacial CBCT: a systematic review	21	7.00	1.18

Top Cited Countries

**Figure 2:**
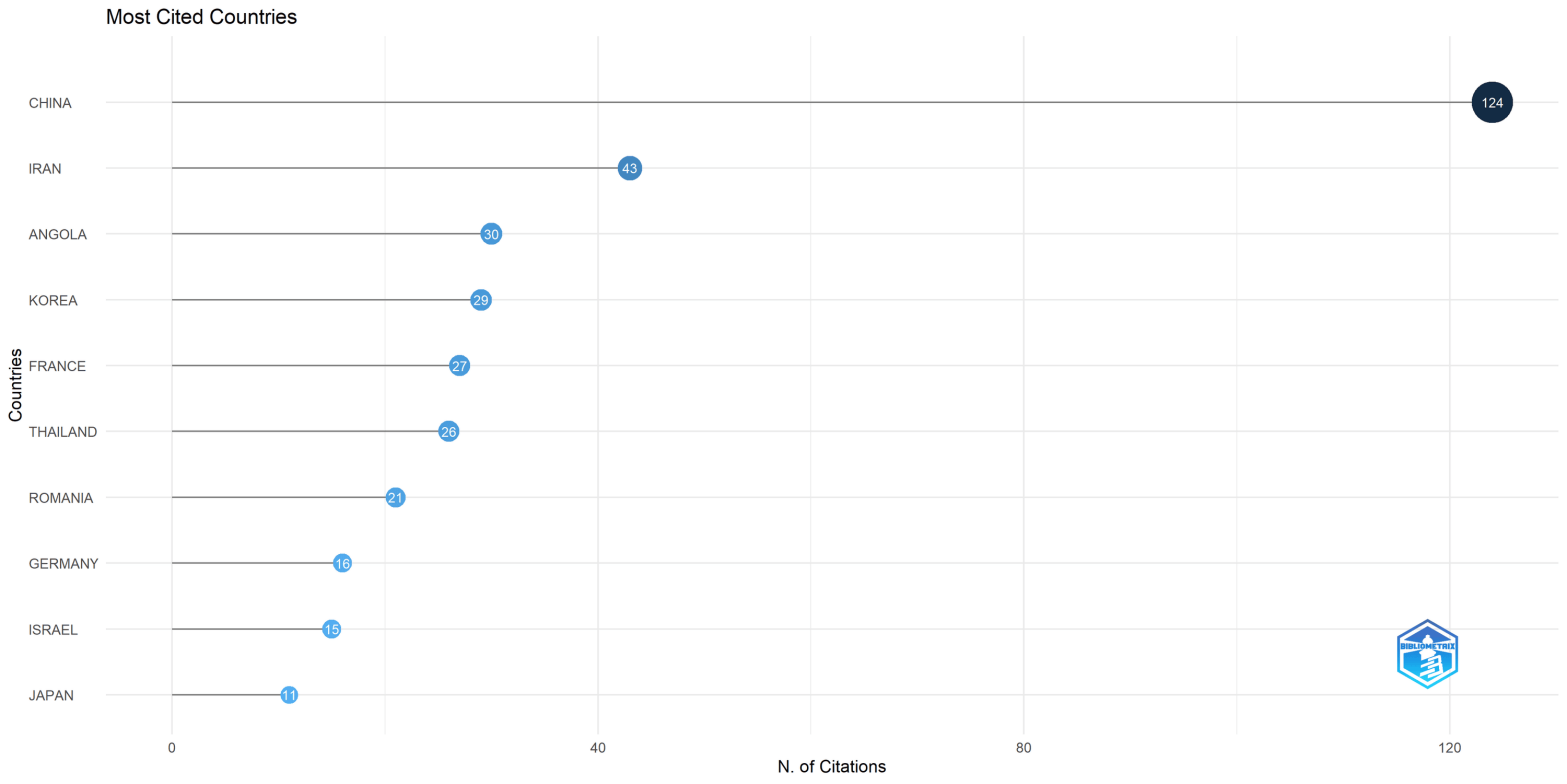
Most Cited Countries

**Figure 3:**
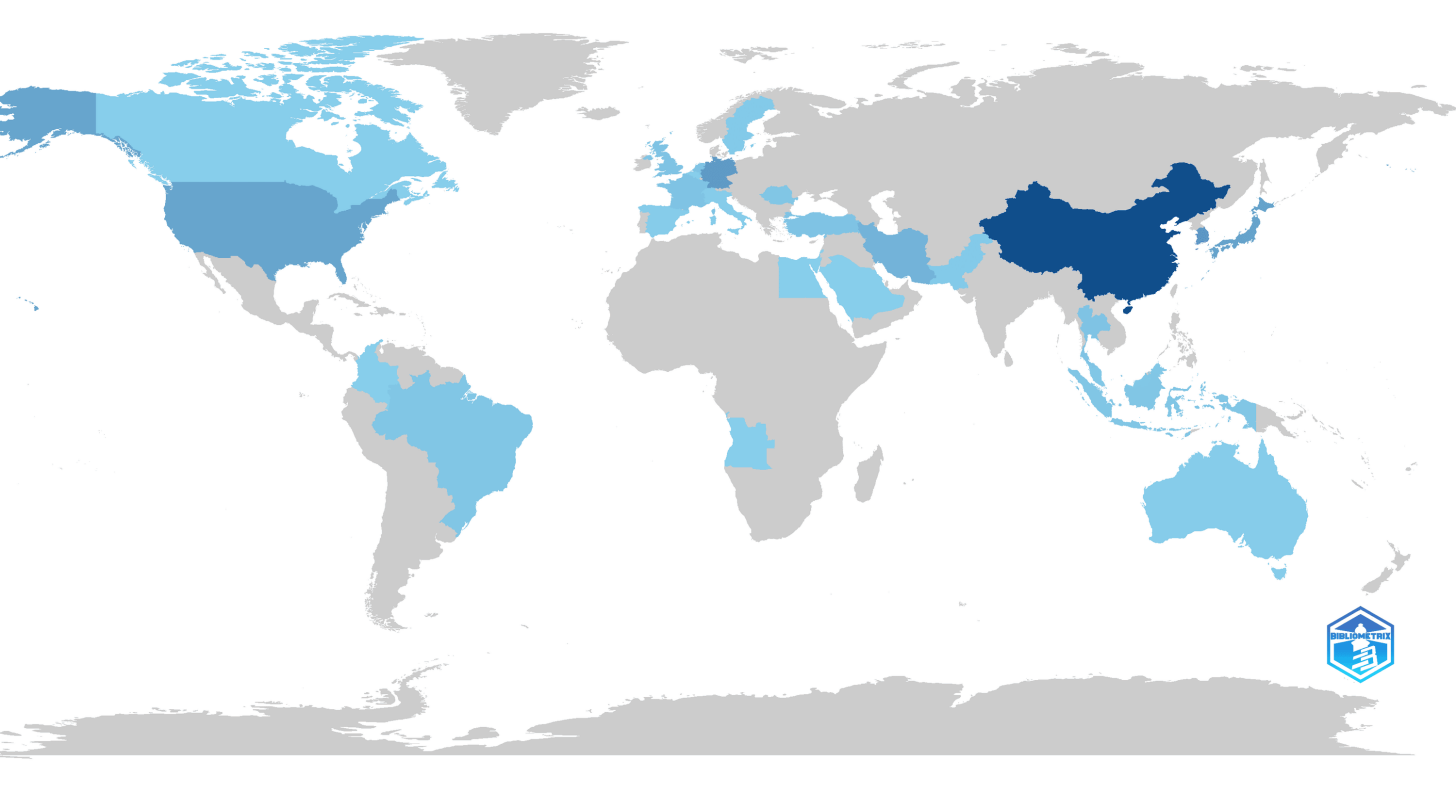
Country Scientific Production

The bibliometric analysis conducted using Biblioshiny highlights China and Iran as leading contributors to AI-based facial bone fracture detection research. China emerges as the most influential, with 124 citations, followed by Iran, which garnered 43 citations. These countries have demonstrated significant leadership and scholarly impact in advancing AI applications within this specialized field (Figures 2, 3).

Institutions With the Most Relevant Articles

The bibliometric analysis revealed that Shanghai Jiao Tong University leads in research output, with 15 articles focused on AI applications in facial bone and fracture detection, particularly from the Department of Plastic and Reconstructive Surgery at Shanghai Ninth People's Hospital. Other institutions with significant contributions include the Department of Cranio- and Maxillofacial Surgery at University Hospital Regensburg in Germany and the Division of Information Science at the Nara Institute of Science and Technology in Japan, each with nine publications (Table 2).

**Table 2:** Institutions With the Most Relevant Articles

Affiliation	Articles
Department of Plastic and Reconstructive Surgery, Shanghai Ninth People’s Hospital, School of Medicine, Shanghai Jiao Tong University	15
Department of Cranio- and Maxillofacial Surgery, University Hospital Regensburg, Franz-Josef-Strauß-Allee 11, 93053 Regensburg, Germany	9
Division of Information Science, Nara Institute of Science and Technology, Nara, Japan	9
Oxford University Hospitals NHS Foundation Trust, Oxford, UK	8
Maxillofacial Surgery Unit, Department of Medical Biotechnologies, University of Siena, 53100 Siena, Italy	7

Keyword Analysis

**Figure 4:**
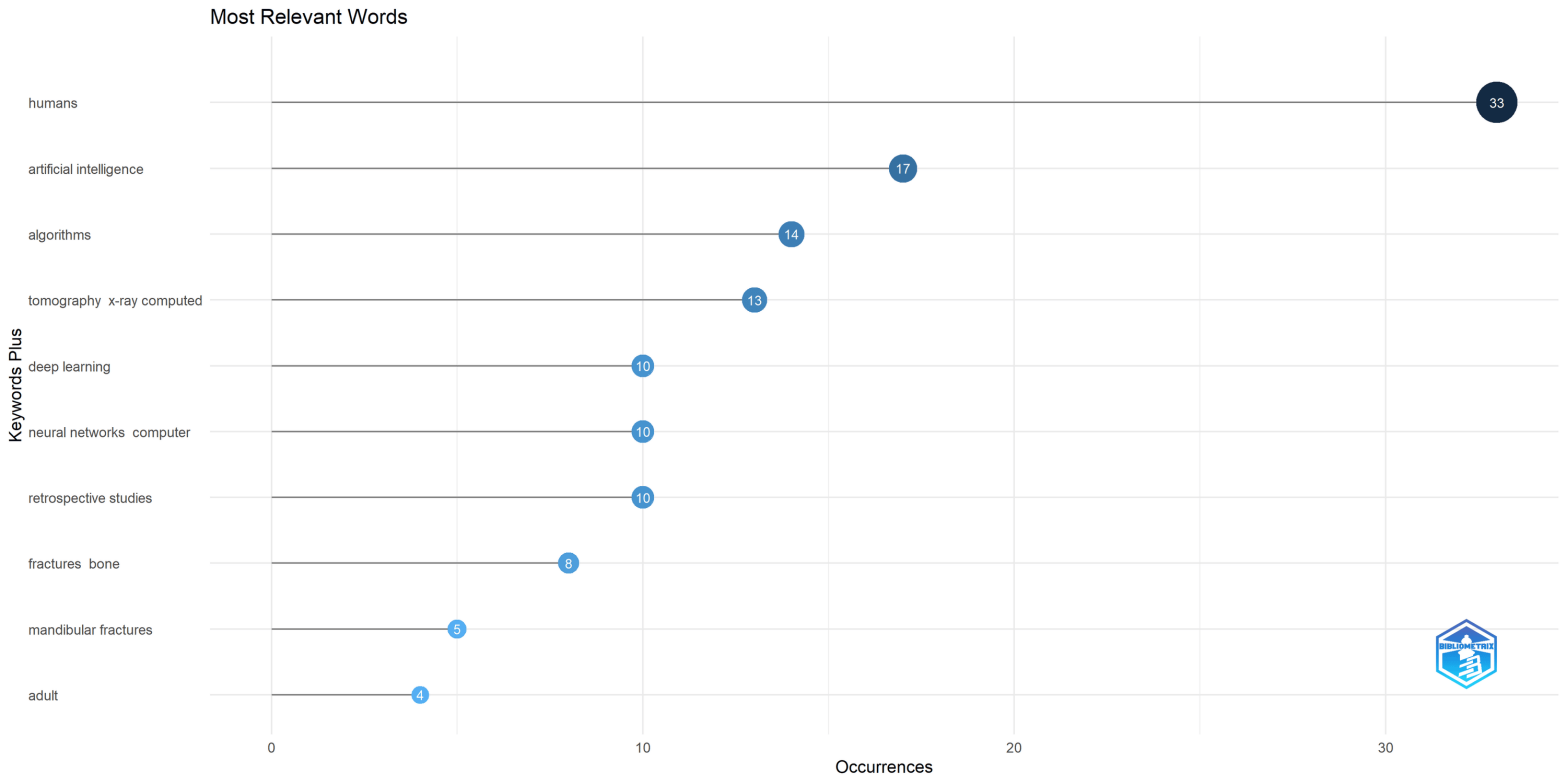
Most Relevant Keywords

**Figure 5:**
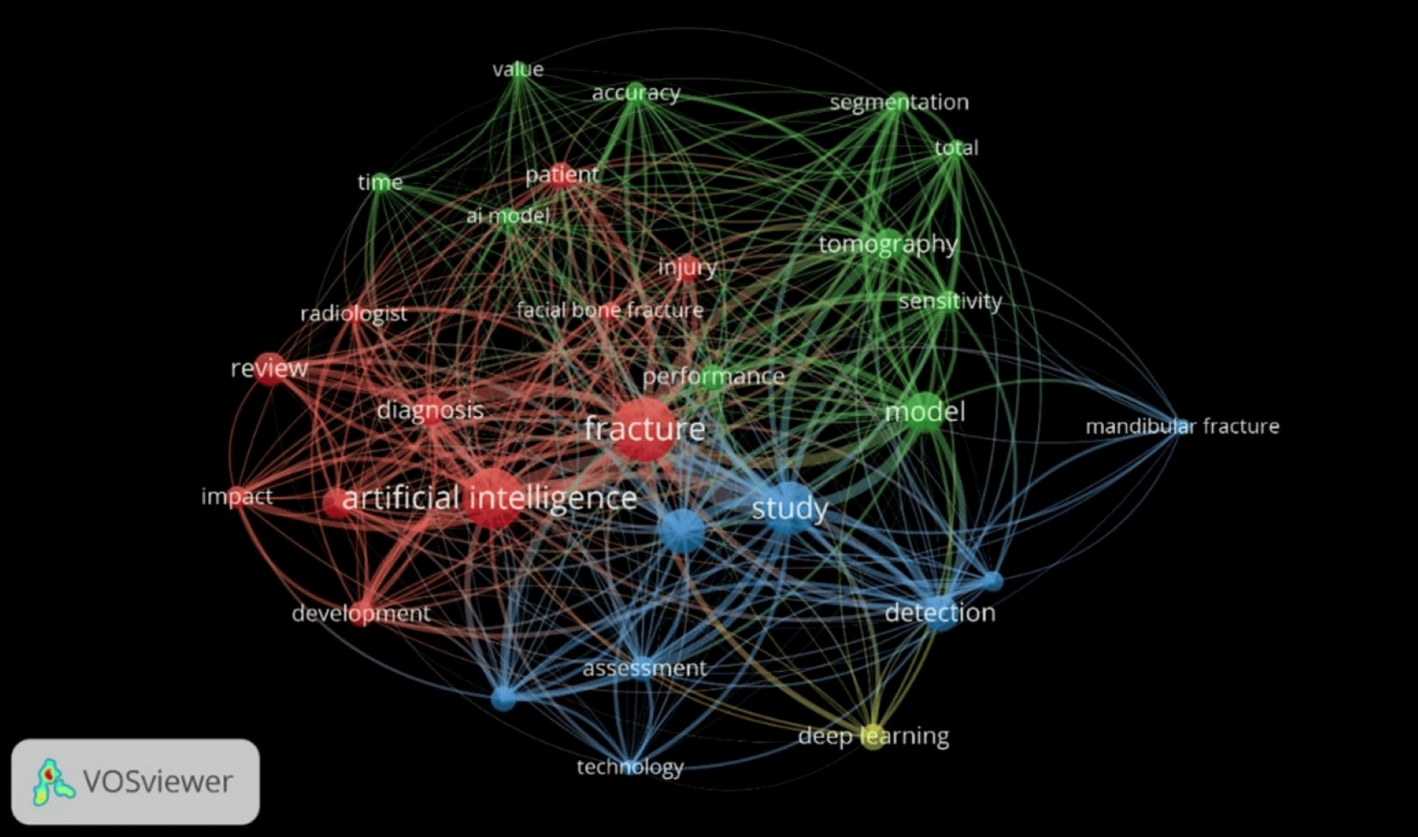
Keyword Analysis Done With VOSviewer

An analysis of the 10 most pertinent keywords revealed “humans” as the predominant term, cited 33 times. Notably, "artificial intelligence" also featured prominently, with a total of 17 occurrences (Figures 4, 5).

Frequency of Words Used Over Time

**Figure 6:**
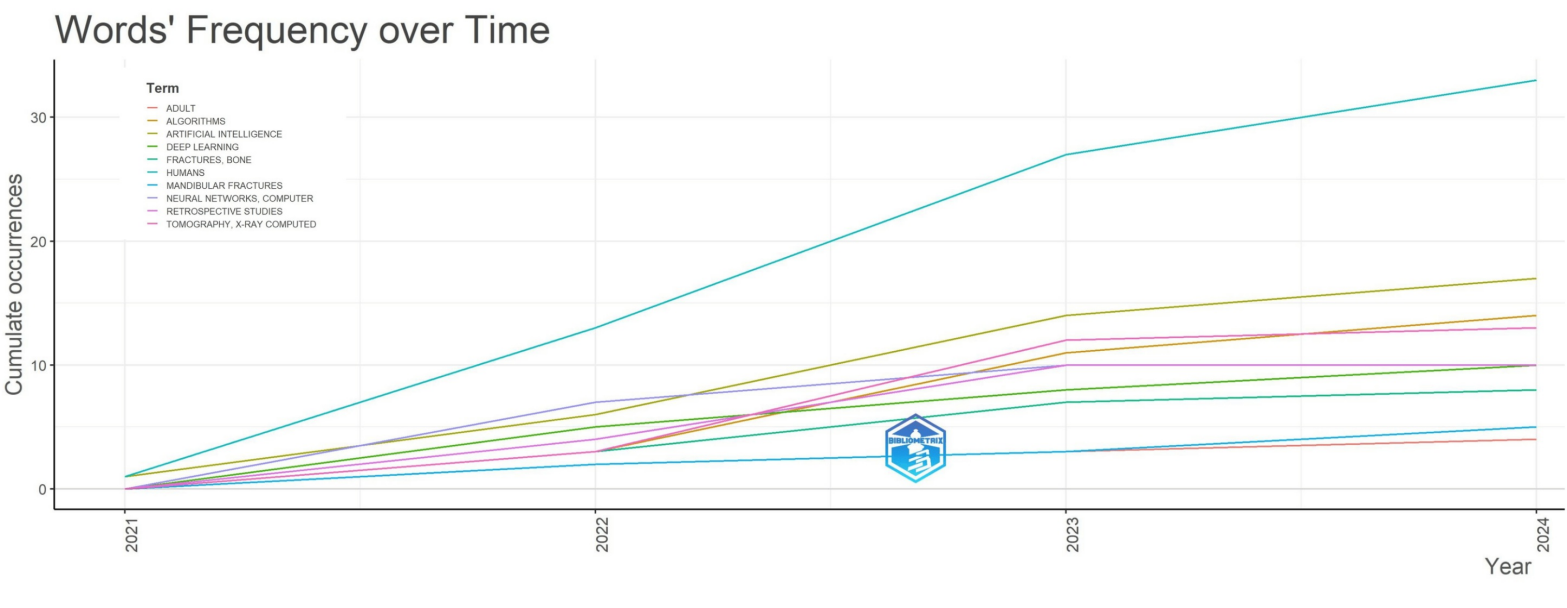
Word Frequency Over Time

The figure below showcases the top 10 most commonly used terms in research on AI for facial bone fractures. These keywords reflect the core topics and key areas of interest that researchers are focusing on within this growing field (Figure 6).

Most Relevant Sources

**Figure 7:**
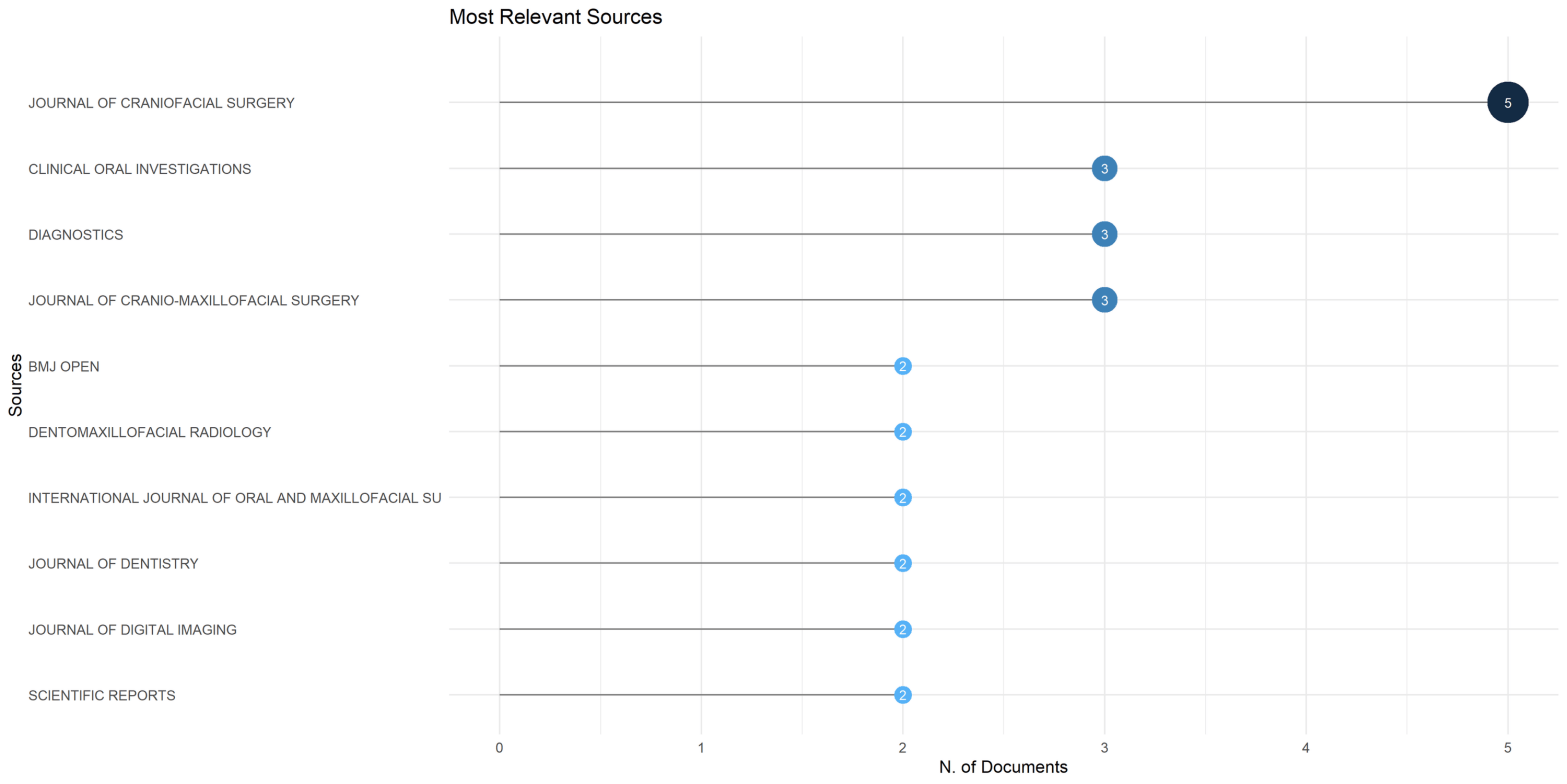
Most Relevant Sources

Based on the bibliometric analysis of the top 10 sources with the most relevant articles on AI on facial bone fractures, the most relevant source is the *Journal of Craniofacial Surgery* and *Clinical Oral Investigations* (Figure 7).

Sources' Production Over Time

**Figure 8:**
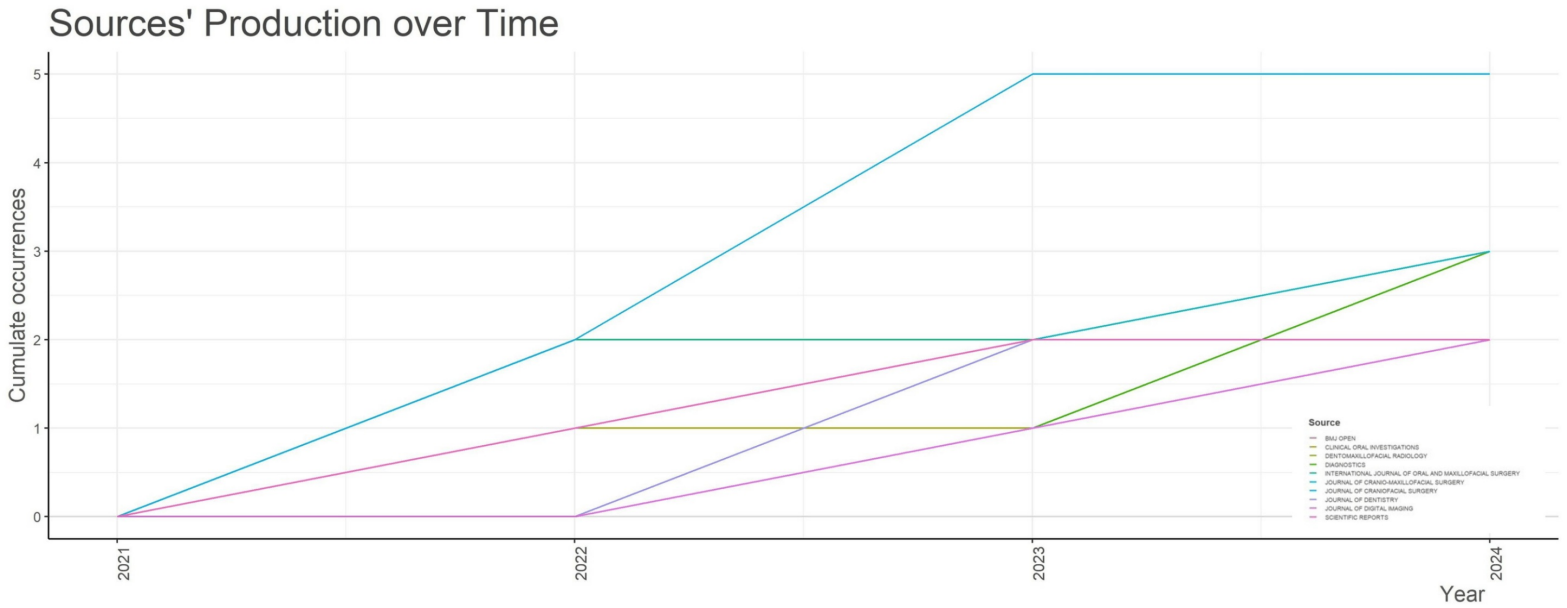
Sources' Production Over Time

According to the bibliometric analysis, the sources’ production from 2021 to 2024 demonstrated a notable increase in output over time. There has been a notable increase in sources like *BMJ Open* since 2022 (Figure 8).

Authors' Local Impact

The authors listed have made notable contributions to the emerging field of AI applications in facial bone fracture, particularly in the past two years, beginning in 2022. Although relatively new in this area, they have already demonstrated a significant scholarly impact. For instance, authors such as Jantana P, Limprasert W, and Suebnukarn S exhibit comparable citation metrics, including h-index and g-index values, reflecting their growing influence and recognition within the academic and clinical communities. Their work is gaining traction, as evidenced by consistent citation patterns, indicating the relevance and applicability of their research in advancing both AI-driven technologies and clinical practice in craniofacial diagnostics and treatments (Table 3).

**Table 3:** Authors' Local Impact h-index: this index measures a researcher's productivity and citation impact by defining h as the number of publications that have at least h citations each. For example, an h-index of 15 means the researcher has 15 papers with at least 15 citations each g-index: the g-index enhances the h-index by taking into account the total number of citations. It is defined such that a researcher with a g-index of g has published at least g papers that, together, received a minimum of g² citations. This approach places greater emphasis on highly cited papers m-index: this time-normalized metric reflects the average increase in a researcher’s h-index per year since their first publication. It is calculated as the h-index divided by the number of years since the researcher’s first publication, allowing comparison between researchers with different career lengths​ [15] TC: total number of citations; NP: number of publication; PY: publication year

Author	h-index	g-index	m-index	TC	NP	PY start
Jantana P	2	2	0.667	57	2	2022
Limprasert W	2	2	0.667	57	2	2022
Suebnukarn S	2	2	0.667	57	2	2022
Vicharueang S	2	2	0.667	57	2	2022
Warin K	2	2	0.667	57	2	2022
Hung KF	2	2	0.667	55	2	2022
Yeung AWK	2	2	0.667	55	2	2022
Wang X	2	3	0.667	40	3	2022
Bai H	1	1	0.333	38	1	2022
Ding P	1	2	0.333	38	2	2022

Discussion

AI-driven solutions revolutionize the area of radiology by enhancing the accuracy and efficiency of facial bone fracture diagnoses with the help of processed radiographic images. Using convolutional neural networks (CNNs), AI can recognize complex patterns within medical images that a human observer might overlook, potentially leading to a faster and more accurate detection of fractures. However, the application of AI in clinical settings poses a challenge. Generalization of the model across different populations and imaging environments is often difficult and usually degrades the performance of the AI outside controlled conditions. Therefore, it is critical to evaluate AI models locally to see if they work correctly with the specific patient data and equipment [16,17].

Moreover, for "black box" nature AI algorithms, interpretability is limited, and due to clinician hesitation, such algorithms are not used with full potential, especially in critical cases. Standardizing the performance metrics and reports may improve consistency and reliability across different studies and clinical sites. In case these challenges are overcome, AI has great potential to support radiologists in case management and sustaining high diagnostic accuracy concerning patient care for the treatment of fractures [18].

AI is rapidly and mainly transforming the world of dental-maxillofacial imaging quickly in the diagnosis and treatment of facial bone fractures. For such complex injuries by nature, this would mean that AI delivers a colossal potential input into the diagnoses with increased accuracy and speed, thus most naturally improving results for patients.

One such prominent study is Wang et al.'s work [5], in which he used the power of a deep CNN to detect and classify mandibular fractures from the CT scan. This work was published in *Clinical Oral Investigations*, where it received a lot of attention with 38 citations. This paper clearly expresses how these AI models can work on the images for fracture identification and classification without human error and hasten diagnoses in trauma cases [5]. In like manner, Hung et al. [6] contributed to this bourgeoning trend by publishing a paper titled "Personalized dental medicine and AI's relevance in dentomaxillofacial imaging." This was published in *Dentomaxillofacial Radiology*, stating how AI contributes to patient-centered care. It has already been cited 32 times and obviously presents an impact of the paper [6]. Warin et al. further made significant contributions to the evaluation of deep CNN models for the detection of mandibular fractures from panoramic radiographs. Panoramic radiography is one of the most common radiological procedures in dentistry. However, the injuries are difficult to detect due to a lack of depth in the imaging modality. But AI has now been thrown into the mix and seems to have made some promise regarding accuracy improvement of detection when it comes to fractures as the current article in the International *Journal of Oral and Maxillofacial Surgery* is cited 31 times [7]. Furthermore, Fatima et al. [8] in *Healthcare* have broadened this perception regarding AI in dentistry, with its current application and going forward. With 30 citations, this paper underlines the fact that AI has tremendous capacity in diagnostics, treatment planning, and even preventive care, hence marking a turning point regarding our approach to dental care [8].

In trauma settings, fractures need to be rapidly and accurately detected. Canoni-Meynet et al. [9] of *Diagnostic and Interventional Imaging* reviewed the role of AI applications in fracture detection during the routine practice of radiologists working with trauma emergencies. The study, cited 27 times, proved that AI-assisted tools are efficient in improving the rate of fracture detection where real-time help is being provided to the radiologists, and there is less chance of missed diagnosis under time-pressure situations [9].

AI integration into medical practice has heightened several ethical concerns. Rokhshad et al. discussed the ethical dilemmas posed by AI applications in oral and maxillofacial cosmetic surgery in their narrative review for the journal *Maxillofacial Plastic and Reconstructive Surgery*. A citation of 26 times underscores the importance of ethical guidelines surrounding AI integration, providing a safeguard against overlooking technological leaps that might compromise patient care [10]. A related work by Warin et al. [11] demonstrated in *Scientific Reports* that the CNN-based models for the detection and classification of maxillofacial fractures directly from the CT images may further be useful. Being referred 26 times, this work also underlines the utility of AI for the diagnosis of craniofacial trauma, as the right and prompt diagnosis is the key to such applications [11].

Not only mandibular or maxillofacial fractures, AI is diversified for the detection of nasal fractures as well. In the journal *Sensors*, Seol et al. published 3D deep learning models for diagnosing nasal fractures in 2022. This study, with 23 citations, indicates that AI can be used effectively through all the structures of the face and is, thus, a very useful tool to be added to the holistic management of facial trauma [12]. Yet another important contribution of Hung et al. in *Clinical Oral Investigations* is research into the possible ability of AI algorithms to change into maxillofacial radiology, thus driving AI further to catalyze enhanced diagnostic accuracy in practice [13]. Finally, Mureșanu et al. published an original contribution to *Oral Radiology*, namely, a systematic review titled "Artificial intelligence models for clinical usage in dentistry with a focus on dentomaxillofacial CBCT: a systematic review" [14]. The review has been cited 21 times up to date and provided the analytical overview of the potential strengthening that AI might bring to enhance cone-beam CT (CBCT) imaging for accuracy and effectiveness in interpretation and further simplify the process of diagnosis in challenging cases [14].

Taken together, these studies describe how AI changes the landscape of facial bone fracture detection, as well as more broadly in maxillofacial surgery. From improved diagnostic accuracy to how AI could be seamlessly integrated into the everyday workflow of clinics, there is a vast potential, exciting enough at any rate, to galvanize the present research and investigations in this field.

Advancements

The use of AI, deep learning, and optimization strategy algorithms has greatly improved the diagnosis and treatment of facial fractures in clinical settings. These advanced techniques enhance the accuracy and speed of fracture detection and allow for personalized assessments of patient's facial structures through medical imaging data.

It explored all the aspects of the mandible from the AI-based non-linear dynamics and network theory point of view to reveal subtle differences and similarities that are inherent among the different mandibles through CT images. For the first time, it presented the use of fuzzy recurrence plots (FRPs) and geostatistics for the analysis of radiographic features. The characteristic mandible patterns in male and female patients along with the difference that exists between the spatial autocorrelation and the topologies in networks can enhance the forensic identification as well as support clinical decision-making during the reconstruction of the mandible. The next AI-based techniques will have the potential to increase accuracy as well as speed in analyzing mandible morphology and could potentially be used as a stepping stone for applications in forensic science and precision medicine [19]. Warin et al. applied the algorithms of deep learning specifically the faster region-based CNN (R-CNN) and RetinaNet on CT images to develop detectors for midface fractures. Among the models tested, the two-stage faster R-CNN produced better performance than RetinaNet because it maintained an average precision of 0.79 and an area under the curve (AUC) of 0.80. By one giant leap, it has stepped forward the area of quick and accurate diagnosis in midfacial fractures, assisting the problems during planning stages of preoperative care together with reducing the chances of misdiagnosis that might eventually lead to poor patient outcomes. Such AI models are highly useful in clinical practice since they provide tools for aiding in the management of trauma and enhancing patient recovery because of improved fracture detection using autonomous detection [20]. Hashem and Hassanein developed the metaheuristic firefly algorithm integrated with multi-layered associative neural networks for jaw fracture classification. So, by fitting the chosen features at the optimal level and training neural networks, they achieved astonishing recognition accuracy of 98.6% of jaw fracture anomalies. Thus, the overall system based on this conception of filtering out inconsistent data and optimizing the improvement of classification with AI can be used to bring an awful increase in the achievement of early diagnosis of jaw fractures, which means a better outcome for clinical practice. An algorithm that can be said to be metaheuristic for selecting features has recently been presented to hasten the diagnosis process and even the accuracy in the discovery of fracture, thereby lowering post-trauma complications [21].

Future of detection of maxillofacial fractures with AI

There are promising futures for AI in facial bone fracture detection, due to the advancements of deep learning models and improved medical imaging technologies that would provide doctors with quicker and more accurate diagnoses [22]. The current complex AI models, such as CNNs, are becoming increasingly sophisticated at fracture detection as well as the irregularity of bones through subtle pattern analysis of CT and X-ray images, thus lessening errors in diagnosis and promoting better clinical outcomes [23]. As the AI systems become better, they are to integrate very smoothly with the health workflow to support real-time assessment and allow physicians to make more prompt and accurate decisions [24]. The models will in the future embrace explainable AI (XAI) principles. It ensures that clinicians can understand how the AI-generated diagnoses have come to be, instills confidence, and thereby helps clinicians embrace the model at the point of clinical practice [25].

Challenges in AI in maxillofacial fracture

One of the primary challenges in utilizing AI for facial bone fracture detection is the reliance on large volumes of high-quality, annotated datasets. AI models, especially those using deep learning, require extensive and precisely labeled data to ensure accuracy and generalizability. However, obtaining such data is challenging due to privacy concerns, variations in imaging quality, and limited access to annotated medical images, which can reduce the robustness of AI applications across diverse patient populations [26]. Additionally, variability in imaging modalities further complicates the development and application of AI in this area. While CT is often the gold standard for bone visualization due to its detailed imaging, it exposes patients to high radiation levels, raising concerns, especially for younger patients and repeat imaging needs [27]. Alternative modalities like CBCT offer lower radiation exposure but may lack the same level of detail necessary for accurate fracture detection, and MRI, although beneficial for soft tissue analysis, is generally less effective for assessing bone structure [28]. This diversity in imaging techniques necessitates that AI models be adaptable to varying image qualities and formats, requiring extensive model training and validation across different modalities and clinical conditions. Furthermore, AI applications in clinical practice face obstacles related to model interpretability and clinician trust, as many AI algorithms operate as "black boxes," making it challenging for healthcare providers to understand or verify the decision-making process [26]. These challenges underscore the importance of developing standardized datasets, improving model transparency, and fostering multi-disciplinary collaboration to advance the clinical integration of AI in facial bone fracture detection.

## Conclusions

There is a significant opportunity for AI to enhance medical diagnostics in fracture and maxillofacial surgery. AI-based image analysis using CNNs can match or surpass human expertise, leading to better diagnostic accuracy and faster treatment decisions. Although AI applications in maxillofacial surgery are still developing, they hold promise for optimizing trauma care and reconstruction. Future uses will likely incorporate advanced imaging tools, like CT and MRI, to improve surgical planning and post-operative assessments. In emergency departments, AI will enhance fracture diagnosis, reduce errors, and help ease the burden on the healthcare system.

Despite these advantages, several issues require clinical validation, and thorough assessments of AI systems across various healthcare environments are crucial. By integrating advanced imaging techniques, we can address ethical concerns surrounding AI usage. Overall, AI has the potential to transform medical diagnostics and significantly improve patient care.
